# The effects of dietary fat on gut microbial composition and function in ovarian cancer

**DOI:** 10.21203/rs.3.rs-5904007/v1

**Published:** 2025-02-07

**Authors:** Mariam M. AlHilli, Naseer Sangwan, Alex Myers, Surabhi Tewari, Daniel J. Lindner, Gail A.M. Cresci, Ofer Reizes

**Affiliations:** Cleveland Clinic; Cleveland Clinic; Cleveland Clinic; Cleveland Clinic Lerner College of Medicine of CWRU; Cleveland Clinic; Cleveland Clinic Lerner College of Medicine of CWRU; Cleveland Clinic

## Abstract

**Objectives:**

The gut microbiome (GM) is pivotal in regulating inflammation, immune responses, and cancer progression. This study investigates the effects of a ketogenic diet (KD) and a high-fat/low-carbohydrate (HF/LC) diet on GM alterations and tumor growth in a syngeneic mouse model of high-grade serous ovarian cancer (EOC).

**Methods:**

Thirty female C57BL/6J mice injected with KPCA cells were randomized into KD, HF/LC, and low-fat/high-carbohydrate (LF/HC) diet groups. Tumor growth was monitored with live, in vivo imaging. Stool samples were collected at the time of euthanasia and analyzed by 16SrRNA sequencing and shotgun metagenomic sequencing was performed to identify differential microbial taxonomic composition and metabolic function.

**Results:**

Our findings revealed that KD and HF/LC diets significantly accelerated EOC tumor growth compared to the LF/HC diet in a xenograft model. GM diversity was markedly reduced in KD and HF/LC-fed mice, correlating with increased tumor growth, whereas LF/HC-fed mice showed higher GM diversity. Metagenomic analyses identified distinct alterations in microbial taxa including *Bacteroides*, *Lachnospiracae bacterium*, Bacterium_D16_50, and *Enterococcus faecalis* predominantly abundant in HF/LC-fed mice, *Dubsiella_newyorkensis* predominantly abundant in LF/HC-fed, and KD fed mice showing a higher abundance of *Akkermansia*and *Bacteroides*. Functional pathways across diet groups indicated polyamine biosynthesis and fatty acid oxidation pathways were enriched in HF/LC-fed mice.

**Conclusions:**

These results highlight the intricate relationship between diet, the gut microbiome, and tumor metabolism. The potential role of dietary interventions in cancer prevention and treatment warrants further investigation.

## INTRODUCTION

The gut microbiome (GM) houses a diverse and rich cohort of bacteria that play a central role in regulating metabolism, inflammation, and immune responses ^1–3^. The intricate relationship between the GM and the host is now understood to play a crucial role in maintaining physiological balance and contributing to disease^4–6^. The GM directly impacts carcinogenesis through its interaction with diet, genetics, lifestyle, and environmental factors ^7,8^. Metabolites produced by GM species, such as bile acids, have pro-inflammatory effects that contribute to DNA damage, genomic instability, and oncogenic signaling ^8,9^. More importantly, decreased GM diversity is strongly associated with adverse treatment outcomes and poor responses to therapy in cancer patients. Understanding how dietary intake impacts GM and cancer progression is essential for exploring therapeutic opportunities targeting dietary interventions in cancer prevention.

Diet is a predominant factor that directly influences the GM composition, and acute dietary changes produce rapid and sustained changes in GM composition and function ^6,10^. For instance, under obesogenic dietary conditions, bile acids produced by the *Firmicutes* phylum have oncogenic effects. However, feeding a high-fiber diet or a Mediterranean-style diet yields short-chain fatty acid (SCFA) production by GM which is associated with tumor suppressive benefits ^11,12^. A high-fat diet shifts the gut microbiome composition to one with an elevated Firmicutes to Bacteriodetes ratio thus disrupting homeostasis and increasing inflammation while reducing the availability of beneficial SCFA^13^.

Poor diet quality and high fat intake strongly correlate with inflammation and adverse oncologic outcomes in many cancers, including ovarian cancer ^14,15^. However, there remains uncertainty about the impact of dietary fat concentration and saturation on cancer progression. For instance, the ketogenic diet (KD) consisting of 80–90% of energy as fat with very low carbohydrate concentration has become a popular diet among cancer patients due to the proposed benefits of KD on tumor biology and anti-tumor response ^16–18^. Furthermore, ketone bodies (e.g., beta-hydroxybutyrate) produced on a KD can significantly alter the GM, selectively inhibit microbial growth, and promote a reduced abundance of *Bifidobacterium* and an increased abundance of *Akkermansia* species ^19–22^.

Dietary intake has been increasingly recognized to influence tumor metabolism. For example, tumor cells increase glucose uptake with increased carbohydrate abundance in the diet, potentially fueling tumor growth through the Warburg effect. High-fat diets also alter lipid availability and composition, using fatty acids for energy and tumor growth. We have shown that treatment with KD (90% fat, 0% carbohydrate) in a syngeneic mouse model of epithelial ovarian cancer (EOC) induced tumor growth and caused significant upregulation of fatty acid metabolism ^23^. Prior work has also demonstrated the role of fatty acids and adipocytes in promoting EOC growth and metastasis ^24,25^. Herein, we explore the diet-microbiome-tumor axis in ovarian cancer and elucidate the effects of a high-fat diet on GM alterations and metabolomic readouts under KD and high-fat diet conditions in a syngeneic model of high-grade serous ovarian cancer.

## MATERIALS AND METHODS

### Cell Line

KPCA EOC cell line, kindly provided by Dr. Robert Weinberg at the Massachusetts Institute of Technology, was utilized in these studies. KPCA tumors harbor mutations in *KRAS, P53, CCNE, and AKT2* overexpression mimicking homologous recombination (HR) proficient high-grade serous EOC^26^. KPCA EOC cell lines were cultured in Dulbecco Modified Eagle Medium (DMEM) media containing heat-inactivated 5% FBS (Atlas Biologicals Cat # F-0500-D, Lot F31E18D1) and grown under standard conditions. For luciferase transduction, in short, HEK 293T/17 (ATCC CRL-11268) cells were plated and co-transfected with Lipofectamine 3000 (L3000015 Invitrogen), 3rd generation packaging vectors pRSV-REV #12253, pMDG.2 #12259, and pMDLg/pRRE #12251 (Addgene) and lentiviral vector directing expression of luciferase reporter pHIV-Luciferase #21375 4.5 μg (Addgene). Viral particles were harvested, filtered through a 0.45 μm Durapore PVDF Membrane (Millipore SE1M003M00) and added to each cell line’s culture media. Viral infections were carried out over 72 hours and transduced cells were selected by their resistance to 2 μg/mL puromycin (MP Biomedicals 0219453910).

### Animal Studies

Female C57BL/6J (BL/6) mice were purchased from Jackson Laboratories (Bar Harbor, ME) at 6–8 weeks of age. Experimental animals were housed and handled in accordance with Cleveland Clinic Lerner Research Institute Institutional Animal Care and Use Committee approved protocol.

Thirty C57BL/6J mice were injected intraperitoneally with murine KPCA-luc cells (5 ×10^6^) on day 0. On day 14, mice were randomized to three diet arms: KD, high fat/low carbohydrate diet (HF/LC), and low fat, high carbohydrate (LF/HC) diet. All murine diets were irradiated and provided by Tekland Envigo^27,28^. HF/LC (RD.160239.PWD) diet consisted of 75% fat from Crisco, cocoa butter, and corn oil and 15% carbohydrates, while KD diet (RD. 160153.PWD) consisted of 90% fat from the same sources and 0% carbohydrates. LF/HC diet (TD.150345) consisted of 13% fat and 77%% carbohydrates. Diet macronutrient and micronutrient composition is shown in Table 1. Weekly *in vivo* imaging system (IVIS) was performed between days 14 and 41. Weekly blood glucose and ketone levels were also assessed following a tail vein blood draw using a standard laboratory glucometer (Precision Xtra^®^). Mouse weight was assessed weekly on a standard laboratory scale. At necropsy, plasma (cardiac puncture) and tumor tissue were collected. Fecal pellets were collected at the endpoint for microbial sequencing and analysis.

### Murine Body Condition Score

Mouse wellbeing was assessed directly: while gently restraining a mouse by holding the base of its tail, the observer (blinded to the treatment group) used the thumb and index finger of the other hand to palpate the degree of muscle and fat over the sacroiliac region. A score from 1–5 was given to each mouse weekly following IP tumor cell injection based on previous literature ^29^. The same observer was used for all tests. Based on established IACUC protocols #2018 – 2003, a body composition score of 2 or lower was defined as meeting endpoint criteria for euthanasia.

### Tumor Monitoring by 2D IVIS Imaging

All bioluminescence images were taken with IVIS Lumina (PerkinElmer) using D-luciferin as previously described ^30^. Mice received an intraperitoneal (IP)injection of D-luciferin (Goldbio LUCK-1G, 150 mg/kg in 150 uL) under inhaled isoflurane anesthesia. Images were analyzed (Living Image Software), and total flux was reported in photons/second/cm^2^/steradian for each mouse abdomen. All images were obtained with an automatic exposure.

### 16SrRNA gene amplicon sequencing

Genomic DNA extraction, 16S rRNA gene amplification, sequencing, and bioinformatic analysis were performed as described previously^1–4^. Briefly, raw 16S amplicon sequence and metadata were demultiplexed using split_libraries_fastq.py script implemented in QIIME2 ^31^. Demultiplexed fastq file was split into sample-specific fastq files using split_sequence_file_on_sample_ids.py script from QIIME2. Individual fastq files without non-biological nucleotides were processed using Divisive Amplicon Denoising Algorithm (DADA) pipeline ^32^. The output of the dada2 pipeline (feature table of amplicon sequence variants (an ASV table)) was processed for alpha and beta diversity analysis using phyloseq ^33^, and microbiomeSeq (http://www.github.com/umerijaz/microbiomeSeq) packages in R. We analyzed variance (ANOVA) among sample categories while measuring the α-diversity measures using plot_anova_diversity function in microbiomeSeq package. Permutational multivariate analysis of variance (PERMANOVA) with 999 permutations was performed on all principal coordinates obtained during CCA with the ordination function of the microbiomeSeq package.

### Shotgun Metagenomics sequencing and bioinformatics analysis

Quality control of the metagenomic reads was conducted as described previously^1,5–8^. Briefly, raw reads were processed for low-quality based filtering using Trimmomatic pipeline ^9^. Host-derived reads were excluded by mapping the reads to the reference human genome (version GRCh38.p14) using BBMap software (sourceforge.net/projects/bbmap/). Quality trimmed reads were processed for taxonomic and functional profiling using Metaphlan2 ^10^ and Humann2 ^11^, respectively. Differential feature selection was performed using Fisher’s exact-t-test^12^. We assessed the statistical significance (*P* < 0.05) throughout, and whenever necessary, we adjusted *P*-values for multiple comparisons according to the Benjamini and Hochberg method to control False Discovery Rate^13^ while performing multiple tests on taxa and pathway abundances according to sample types. Differential network analysis was performed at the species level using NetComi set at the following parameters; filtTax=”highestVar”, filtTaxPar = list(highestVar = 40), measure=”sparcc”, normMethod = “mclr”, zeroMethod =”none”, sparsMethod = “none”, dissFunc = “signed”. Eigenvector centrality was used for defining hubs and scaling node sizes. Node colors represent clusters, which are determined using greedy modularity optimization. Clusters have the same color in both networks if they share at least two taxa. Nodes that are unconnected in both groups were removed

### Statistical Analysis

Differential abundance test benchmarking was performed using DAtest package (https://github.com/Russel88/DAtest/wiki/usage#typical-workflow). Briefly, differentially abundant methods were compared with False Discovery Rate (FDR), Area Under the (Receiver Operator) Curve (AUC), Empirical power (Power), and False Positive Rate (FPR). Based on the DAtest’s benchmarking, we selected lefseq and anova as the methods of choice to perform differential abundance analysis. We assessed the statistical significance (P < 0.05) throughout, and whenever necessary, we adjusted P-values for multiple comparisons according to the Benjamini and Hochberg method to control the False Discovery Rate(Benjamini and Hochberg, 1995). Linear regression (parametric test), and Wilcoxon (Non-parametric) tests were performed on genera and species abundances against metadata variables using their base functions in R (version 4.1.2; R Core Team, 2021) (Team, 2021).

### Study Approvals

All murine studies were completed in accordance with the Institutional Animal Care and Use Committee guidelines, approval # 2018 – 2003. All studies utilizing lentiviral particle generation were completed in accordance with the Institutional Biosafety Committee guidelines, approval #IBC0920.

## RESULTS

### KD and HF/LC diets induced the growth of KPCA ovarian tumors.

**Figure 1:**
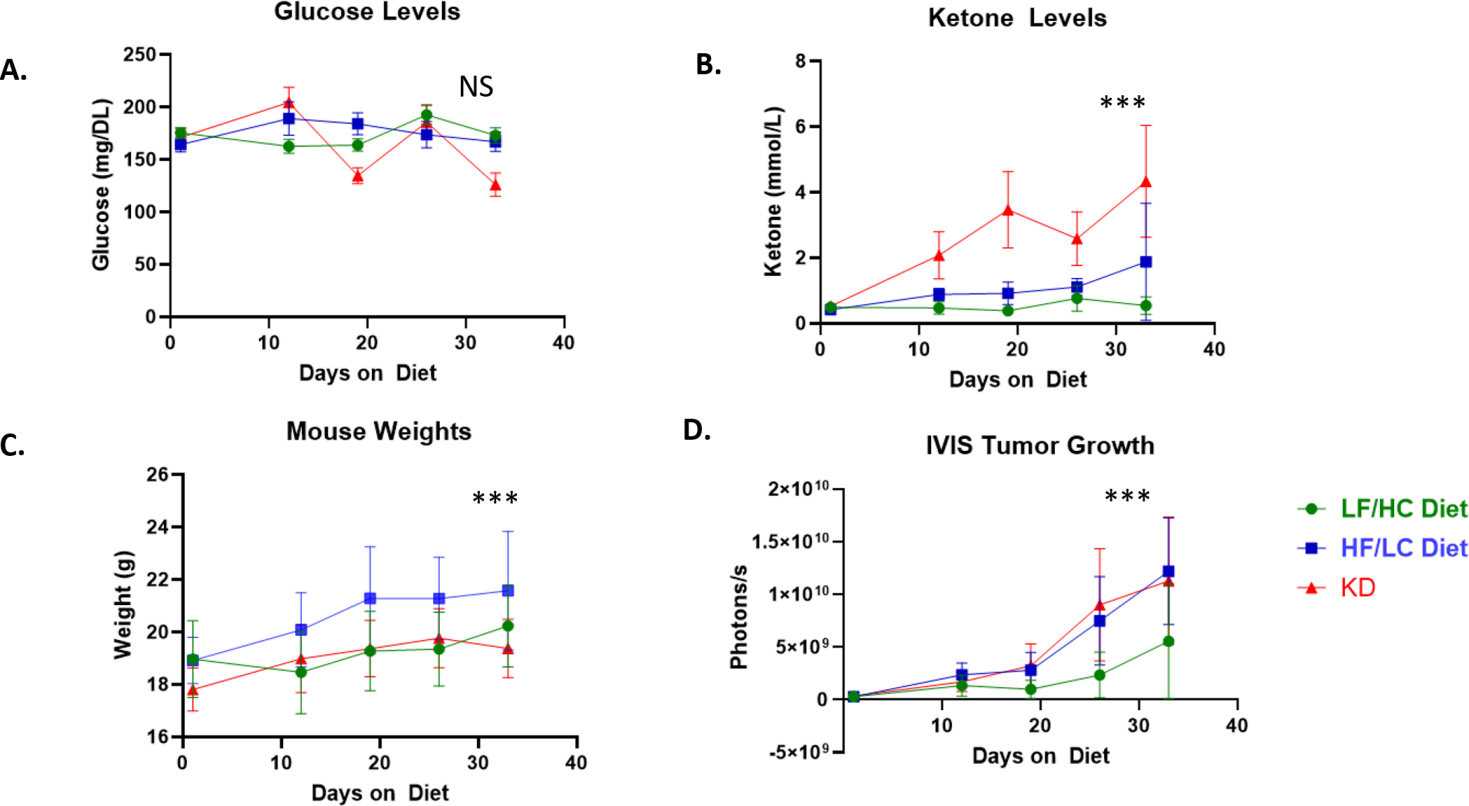
KD and High Fat Diets induced ketones and tumor growth. Ketogenic diet and HF/LC diet in C57Bl/6 mice bearing KPCA tumors induces ketosis and tumor growth (**A**) Weekly circulating glucose levels in each diet arm remain stable throughout the study (**B**) Weekly circulating ketone levels in each diet arm with significant increase in KD fed mice noted (**C**) Mouse weight over the study course between the three diet groups HF/LC diet treated mice show significant increase in body weight compared to KD and LC/HF diet.. (**D**) Increase in tumor growth in mice fed KD and HF/LC diet relative to LF/HC diet fed mice (n = 10 mice per group, ANOVA *** *p* < 0.001, n.s.= not significant).

To investigate the impact of KD vs. HF/LC diet on EOC growth, 30 female C57BL/6J (BL/6) mice were injected intraperitoneally with KPCA Luc cells (5×10^5^). Two weeks after injection, mice were randomized to one of three diet arms (KD, HF/LC diet, and LF/HC diet (10 mice per arm). Glucose levels remained stable throughout the study, with no difference between groups (Fig. 1A). By endpoint, mice treated with KD demonstrated a significant increase in circulating ketone levels compared to HF/LC and LF/HC diet-fed mice, confirming mice were in ketosis (p < 0.001) (Fig. 1B). Body weight was significantly higher in HF/LC diet-fed mice relative to KD and LF/HC diet-fed mice (p < 0.001) (Fig. 1C). Mice fed KD and HC/LC diet showed a marked increase in tumor growth (Fig. 1D) compared to LF/HC diet-treated mice (P < 0.001).

### Decreased gut microbial diversity in HF/LC and KD fed mice compared to LF/HC fed mice.

**Figure 2:**
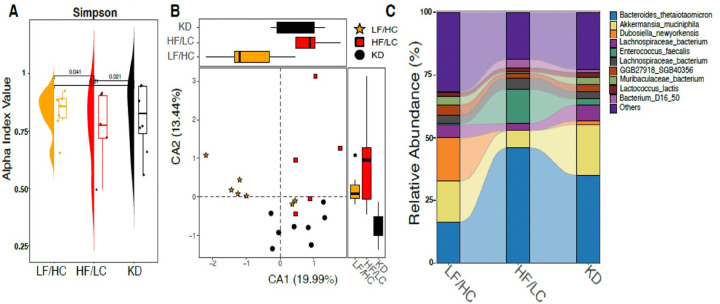
Alpha diversity and relative abundance of bacterial species KD, HF/LC, and LF/HC. (A) Box plot depicting the alpha diversity measured by the Simpson metric for the three groups. The box plot illustrates the distribution of alpha diversity scores within each group, highlighting median values, interquartile ranges, and potential outliers. (B) Principal Component Analysis (PCA) plot representing beta diversity among the three groups. Points on the PCA plot indicate the sample scores for each group, facilitating visualization of the variability and clustering of microbial communities across the different dietary conditions. (C) A stacked flow bar plot shows the relative abundance of microbial taxa across the KD, HF/LC, and LF/HC groups. The plot illustrates the composition of microbial communities, with different colors representing various taxa, allowing for a comparative analysis of community structure among the groups.

Due to the known impact of diet on GM diversity, we sought to characterize differences between GM taxa among diet groups and elucidate alterations in GM species abundance in response to diet. Alpha diversity is shown in Fig. 2A and 2B. Overall, LF/HC diet-fed mice showed the highest alpha diversity while HF/LC and KD-fed mice showed a marked decrease in gut microbial diversity. Figure 2B shows a significant leftward separation in microbial abundance between LF/HC control-fed mice and KD and HF/LC diet-fed mice. The key differentially abundant gut microbial species included *Bacteroides thetaiotamicron*, *Lachnospiraceae bacterium*, Bacterium_D16_50 and *Enterococcus faecalis* predominantly abundant in HF/LC-fed mice Fig. 2C, and *Dubosiella newyorkensis* predominantly abundant in LF/HC-fed mice. KD-fed mice had a higher abundance of *Akkermansia* relative to HF/LC and LF/HC diet fed mice, while *Bacteroides thetaiotamicron* was highly abundant in HF/LC diet relative to the two other groups.

**Figure 3:**
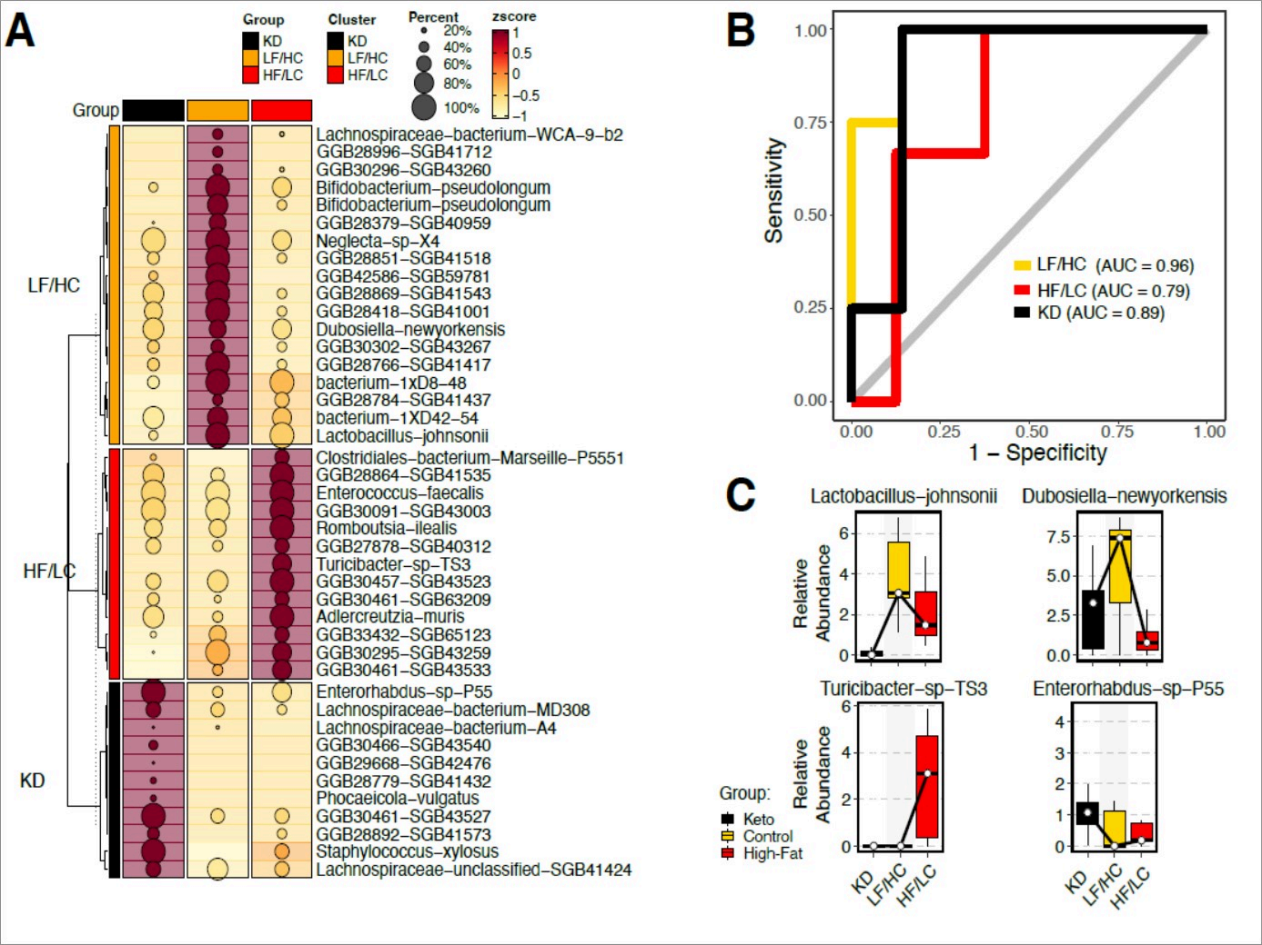
Differential Microbiome taxonomic composition across Groups. **(A)** Heatmap displaying the differentially abundant species across the three groups: KD, HF/LC, and LF/HC. The bubble size in the heatmap corresponds to the fraction of samples in which each species is present, while the color gradient indicates the abundance z-score, with warmer colors representing higher abundance. **(B)** Area Under the Curve (AUC) plot illustrating the specificity (y-axis) versus 1-specificity (x-axis) for the microbiome’s predictive performance across the groups. Each colored line represents a specific group’s performance, demonstrating the microbiome’s discriminative ability to distinguish between them. **(C)** Box plot derived from Tukey’s Honest Significant Difference (HSD) analysis, highlighting the statistical significance of key species identified in the heatmap. This plot assesses the capability of these species to differentiate between the KD, HF/LC, and LF/HC groups.

The associations between gut microbial taxa and diet are illustrated in Fig. 3A. Distinct clustering was noted for each diet group. A strong positive correlation was seen between both *Lactobacillus johnsonii* and *Dubosiella newyorkenesis* and LF/HC diet, while the same species were negatively correlated with HF/LC diet. A similar strong association was also noted between bacterium 1xD8–48, Neglecta, and *Bifidobacterium-pseudolongum*, and LF/HC diet compared to HF/LC diet. Within the HF/LC diet group, a significant positive correlation was noted between several species, including *Clostridial bacterium*, *Enterococcus faecalis*, *Romboustia*, and *Turibacter sp*-TS3. *Enterohabdus-sp-p55*, Lachnospiraceae, *Staphylococcus xylosus*, and *Lachnospiraceae bacterium*-MD308 were significantly associated with KD. Receiver operator curves demonstrate high predictive accuracy for KD taxonomy group (AUC 0.89), HF/LC group (AUC 0.79), and control (AUC 0.98) (Fig. 3B). The statistical significance of the key species *Lactobacillus johnsonii*, *Dubosiella newyorkenesis, Turibacter sp-TS3 and Enterohabdus-sp-p55* is shown in Fig. 3C. As demonstrated, these species show high predictability in distinguishing between each of the diet groups

**Figure 4:**
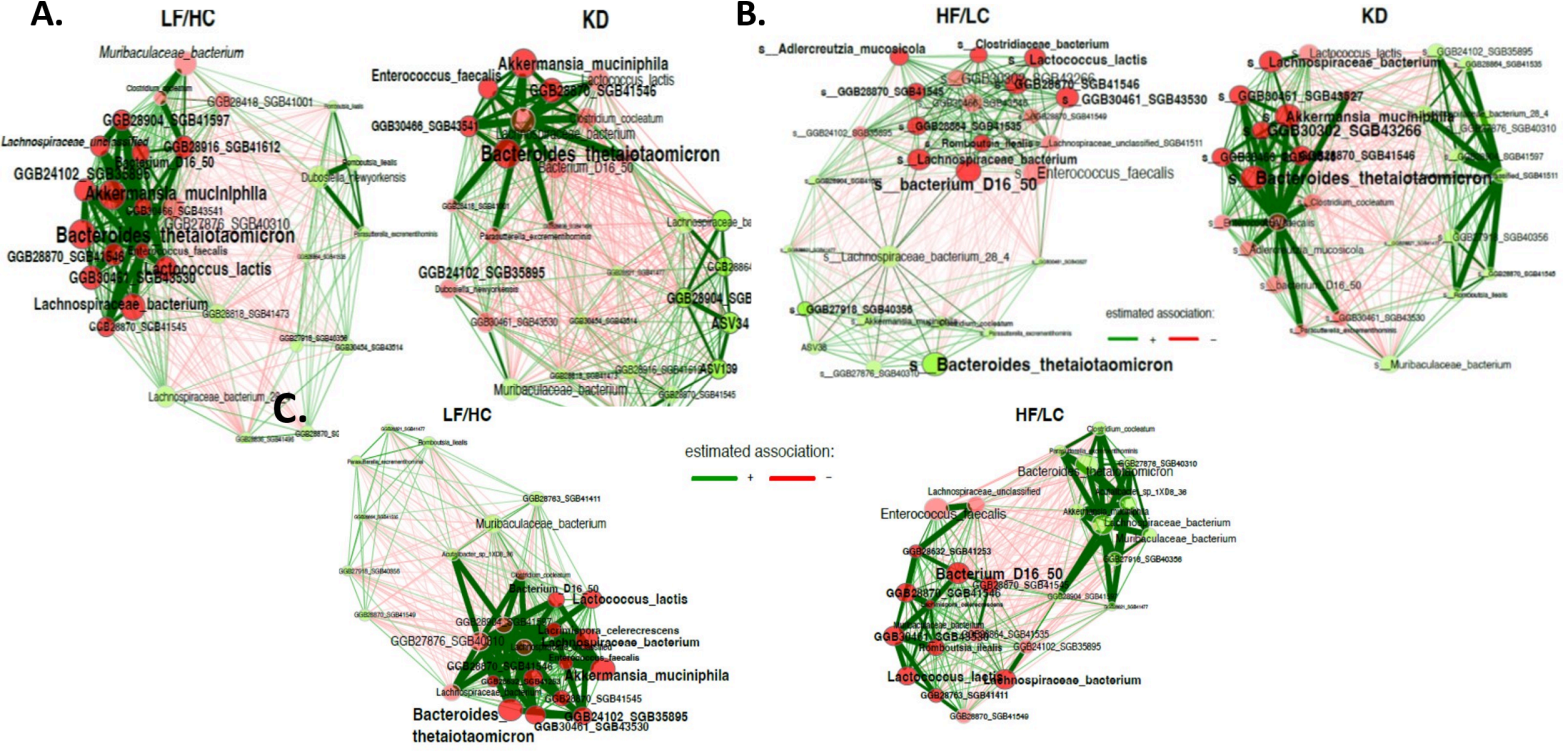
Comparative differential network analysis across diet groups. **(A)** the relationship between LF/HC and KD, **(B)** the connection between HF/LC and KD, and network **(C)** dissimilarity between LF/HC and HF/LC groups, utilizing consistent analytical parameters throughout.

Differential network analysis was used to visually represent functional associations between diet groups, as shown in Figs. 4A–C. Eigenvector centrality was used to define hubs and scaling node sizes, with node size representing relative abundance. Associations between different subject groups were captured utilizing Aitchison’s distance. The analysis is restricted to samples and species with at least 1000 reads, with normalization performed to achieve fraction-based counts. To address the presence of zeros for clr transformation, “multiplicative imputation” is employed. Each dissimilarity matrix is scaled to a range of [0,1] and employs the k-nearest neighbor method (=3) for sparsification. Colors of the nodes indicate clusters derived from hierarchical clustering using average linkage, where clusters sharing the same color have a minimum of 100 nodes in common. Hubs are depicted with bold borders and are defined as nodes with eigenvector centrality surpassing the 99% quantile of the empirical distribution. Edge thickness reflects the degree of similarity, and nodes are arranged closer together based on their compositional similarity, with unconnected nodes excluded from the visualization. Positive correlations are noted in green, and negative correlations are shown in red. Comparisons between HF/LC and KD groups revealed a significant positive association between the following bacterial species in KD: *Bacteroides thetaiotamicron*, *Akkermansia muciniphilia*, *Lachnospiraceae bacterium* and GGB30302 SGB432661. A similar pattern was noted in comparing KD and LF/HC diets. For LF/HC compared to HF/LC diets, significant positive associations between *Bacterium D16* 50, *Lactococcus lactus*, and *Lachnospiraceae bacterium* and HF/LC diet were detected.

### Functional analysis of GM reveals significant metabolic pathway alterations between diet groups.

**Figure 5:**
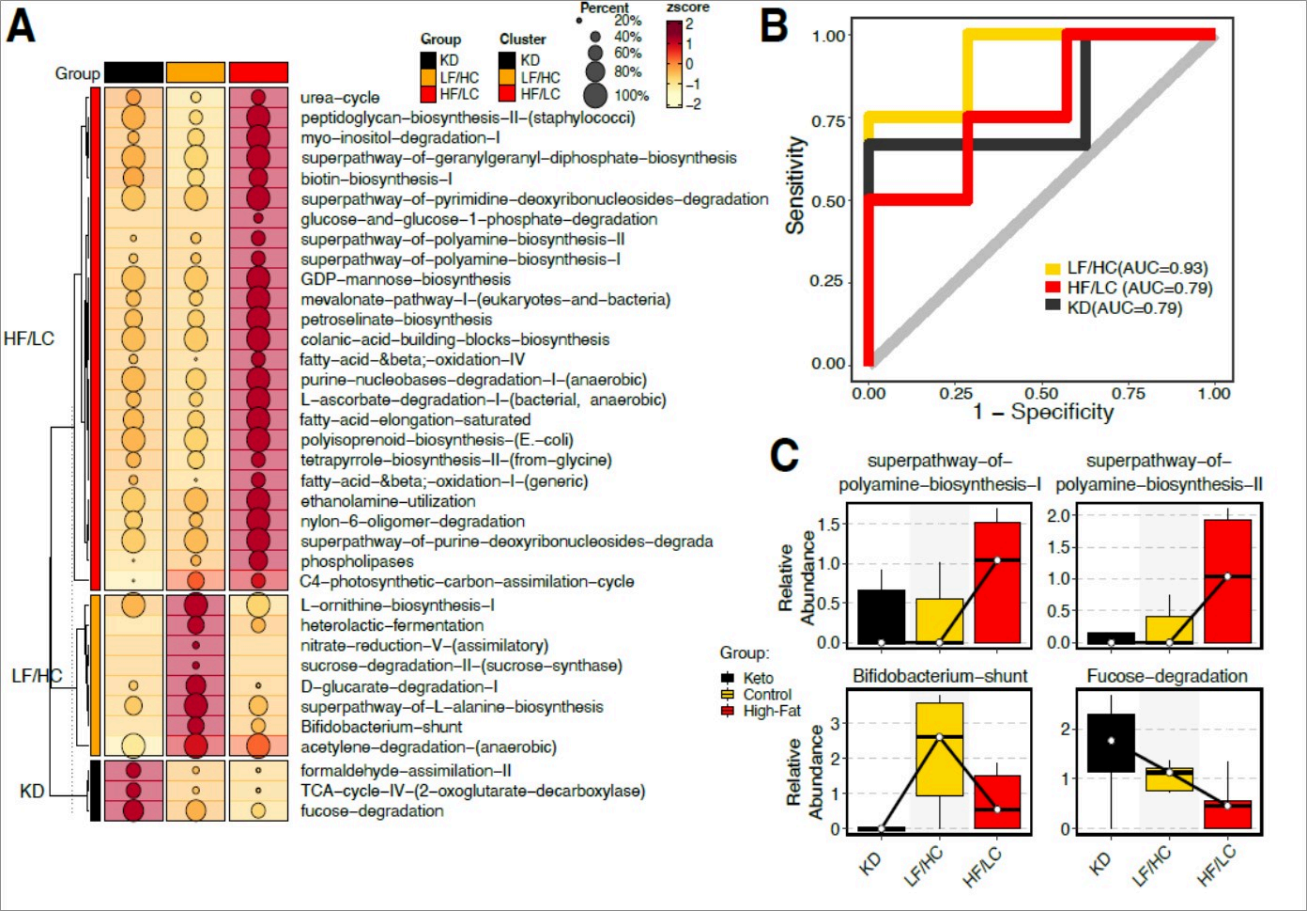
Differential Microbiome functional composition across groups. **(A)**Heatmap displaying the differentially abundant functional pathways across the three groups: KD, HF/LC, and LF/HC. The bubble size in the heatmap corresponds to the fraction of samples in which each species is present, while the color gradient indicates the abundance z-score, with warmer colors representing higher abundance. **(B)** Area Under the Curve (AUC) plot illustrating the specificity (y-axis) versus 1-specificity (x-axis) for the microbiome’s predictive performance across the groups. Each colored line represents a specific group’s performance, demonstrating the microbiome’s metabolic potential to distinguish between them. **(C)** Box plot derived from Tukey’s Honest Significant Difference (HSD) analysis, highlighting the statistical significance of key functional pathways identified in the heatmap. This plot assesses the capability of these pathways to differentiate between the KD, HF/LC, and LF/HC groups.

Functional pathway analysis was performed to identify bacterial metabolic alterations across LF/HC and HF/LC diets. As shown in Fig. 5, a significantly higher abundance of super pathways of polyamine biosynthesis I and II, as well as fatty acid and beta-oxidation pathways and phospholipases, was observed in the HF/LC diet group. Similarly, the fucose degradation pathway was enriched in the KD group, while L-ornithine biosynthesis, the bifidobacterium shunt, the super pathway of L-alanine biosynthesis, and D-gluconate degradation were among the pathways significantly enriched in the LF/HC diet group (Fig. 5A). Receiver operator curves demonstrate high predictive accuracy for functional composition across diet groups (Fig. 5B). Finally, we used Tukey’s Honest Significant Difference (HSD) test (as a post hoc test) to test the statical significance of individual pathways in differentiating all groups. As illustrated in Fig. 5C, the polyamine biosynthesis significantly influenced tumor growth with marked differential abundance between HF/LC and LF/HC and KD groups.

## DISCUSSION

Results of our investigation demonstrate that both KD and HF/LC diets induced significant growth in KPCA EOC tumors compared to the LF/HC diet. Despite stable glucose levels across all groups, KD-fed mice exhibited elevated circulating ketone levels, confirming ketosis, and increased tumor growth. The HF/LC diet similarly promoted tumor growth, highlighting the potential oncogenic influence of high-fat intake regardless of carbohydrate restriction. Our results also demonstrated a generalized increase in gut microbial alpha diversity in mice fed an LF/HC diet and decreased diversity in mice fed an HF/LC and KD diet.

The decrease in alpha diversity correlated with the increase in EOC tumor growth seen in our mouse models. It has been well established in prior studies that a lower gut microbial diversity is strongly associated with adverse treatment outcomes and poor response to therapy in patients with cancer ^15^. The mechanisms that KD and increased dietary fat alter gut microbial abundance are complex and have not been comprehensively defined. One mechanism involves the increase in levels of the ketone body beta-hydroxybutyrate (β-HB) on a KD, which is correlated with a reduced abundance of *Bifidobacterium* ^21^. *Bifidobacteria species* have been shown to have anti-proliferative and anti-apoptotic properties in colorectal cancer, and its abundance was associated with response to therapy in lung cancer-treated mice ^10,22,34^. We indeed showed that mice fed KD had a lower abundance of *Bifidobacteria* which were overrepresented in LF/HC diet-fed mice that had a lower growth rate of EOC than KD-fed mice.

We additionally noted a distinct clustering of bacterial taxa within each diet group, highlighting the discrete yet complex relationship between dietary intake and gut microbial composition. We noted that *Lachnospiracae bacterium*, *Bacterium _D16*_50, and *Enterococcus faecalis* are predominantly associated with HF/LC-fed, and *Dubsiella_newyorkensis* predominantly associated with LF/HC-fed mice. *Dubosiella* is a genus within the family *Lachnospiraceae*, which is part of the Firmicutes phylum. It is the murine homologue of Clostridium, which has a probiotic immunomodulatory effect and is known for producing short-chain fatty acids that contribute to a balanced gut microbiome. On the other hand, increased abundance of *Enterococcus fecalis* has been previously shown to be associated with a high fat diet, which tends to be associated with inflammation, toxin-related damage, and cancer progression. *E. faecalis*, however, plays a controversial role in the development of colorectal cancer ^35^. As demonstrated in the network plots, complex correlations between bacterial taxa and distinct clustering was noted, confirming prior findings demonstrating that a high-fat diet drives changes in gut microbiota with over underrepresentation of beneficial butyrate-producing bacteria ^36^.

Functional shotgun metagenomics analyses were performed to understand the mechanistic implications behind these associations. A critical pathway overrepresented in the HF/LC diet gut microbial analysis was the polyamine biosynthesis pathway. Other significant pathways included fatty acid elongation and fatty acid and beta-oxidation. In contrast, TCA cycle IV and fucose degradation were among the highly overrepresented metabolic pathways in KD. Polyamines play an essential role in cellular growth and differentiation and can influence antitumor immune response and modulate inflammation. Intake of polyamines and dysregulation of polyamine metabolism is a feature of many cancers, including colorectal cancer ^37^. Furthermore, polyamine metabolism was found to regulate immune cells, including tumor-associated macrophages and T cells in hepatocellular carcinoma. Polyamine metabolism is regulated by MYC through the rate-limiting enzyme, ornithine decarboxylase. Moreover, the FDA-approved anti-protozoan drug, α-difluoromethylornithine (DFMO), inhibits ODC activity and induces polyamine depletion, leading to tumor growth arrest ^38^. Herein, we show that polyamine biosynthesis was functionally enhanced by the gut microbiota of HF/LC-fed mice and was predictive of HF/LC dietary intake in mice. Whether this outcome is mediated through the immune microenvironment is imperative to investigate in future studies.

On the other hand, fucose is a methylpentose, which was elevated in KD diet-fed mice stool samples, is found in glycoproteins and glycolipids. It is metabolized from dietary glycans by alpha fucosidase, which is prevalent in gut bacteria. *Bacterium thetaiotamicron*, associated with KD, is proficient at utilizing fucose for energy ^39^. This suggests that fucose degradation by gut microbiota in mice fed KD may play a role in mediating tumor growth. Furthermore, the role of fatty acid beta-oxidation in the progression of tumor growth on HF/LC is noteworthy and has been demonstrated in prior studies ^36^. Data from our group (unpublished) showed that an HF/LC diet was associated with the upregulation of the fatty acid oxidation gene, CPT1A. Fatty acid metabolism dysregulation is a characteristic feature of EOC and correlates with poor survival ^40^. Thus, the effects of HF/LC on tumor growth may be potentially mediated through the utilization of available fatty acids and upregulation of fatty acid metabolism.

Our findings underscore the complex interplay between diet, tumor metabolism, and the gut microbiome. We utilized advanced metagenomic analyses to validate and characterize the associations between diet and gut microbiota, shedding light on the intricate interactions between gut microbiome and metabolome. One of the main limitations of our study, however, is that we could not assess the effects of EOC growth on changes in gut microbial composition, as we did not control for tumor presence in our studies. Further correlations between gut, tumor, and plasma metabolites would solidify the results of our study.

The pro-tumorigenic effects of KD and HF/LC diets raise important considerations for dietary recommendations in cancer patients. While KD has gained popularity for its potential anti-tumor effects, our data suggests it may not be universally beneficial and could potentially fuel tumor growth in specific contexts. These findings contribute to the growing body of evidence on the critical role of diet in cancer progression and the potential for dietary manipulation as a therapeutic strategy.

## Data Availability

The datasets used and/or analyzed during the current study are available from the corresponding author on reasonable request.
